# Number of teeth is associated with all-cause and disease-specific mortality

**DOI:** 10.1186/s12903-021-01934-0

**Published:** 2021-11-08

**Authors:** Yau-Hua Yu, Wai S. Cheung, Bjorn Steffensen, Donald R. Miller

**Affiliations:** 1 Department of Periodontology, Tufts University School of Dental Medicine, One Kneeland Street, Boston, MA 02111 USA; 2 Center for Healthcare Organization and Implementation Research, Edith Nourse Rogers Memorial Veterans Hospital, VA Bedford Health Care System, Bedford, MA USA; 3 School of Public Health, Department of Health Law, Policy and Management, Boston University, Boston, MA USA

**Keywords:** Tooth loss, Edentulism, Mortality, Diabetes, Stroke, Hypertension, Cardiovascular diseases, Femoral neck bone mineral density

## Abstract

**Background:**

Tooth loss has been shown to correlate with multiple systemic comorbidities. However, the associations between the number of remaining natural teeth (NoT) and all-cause mortality have not been explored extensively. We aimed to investigate whether having fewer NoT imposes a higher risk in mortality. We tested such hypotheses using three groups of NoT (20–28,10–19, and 0–9), edentulism and without functional dentition (NoT < 19).

**Methods:**

The National Health and Nutrition Examination Survey in the United States (NHANES) (1999–2014) conducted dental examinations and provided linkage of mortality data. NHANES participants aged 20 years and older, without missing information of dental examination, age, gender, race, education, income, body-mass-index, smoking, physical activities, and existing systemic conditions [hypertension, total cardiovascular disease, diabetes, and stroke (N = 33,071; death = 3978), or with femoral neck bone mineral density measurement (N = 13,131; death = 1091)] were analyzed. Cox proportional hazard survival analyses were used to investigate risks of all-cause, heart disease, diabetes and cancer mortality associated with NoT in 3 groups, edentulism, or without functional dentition.

**Results:**

Participants having fewer number of teeth had higher all-cause and disease-specific mortality. In fully-adjusted models, participants with NoT0-9 had the highest hazard ratio (HR) for all-cause mortality [HR(95%CI) = 1.46(1.25–1.71); *p* < .001], mortality from heart diseases [HR(95%CI) = 1.92(1.33–2.77); *p* < .001], from diabetes [HR(95%CI) = 1.67(1.05–2.66); *p* = 0.03], or cancer-related mortality [HR(95%CI) = 1.80(1.34–2.43); *p* < .001]. Risks for all-cause mortality were also higher among the edentulous [HR(95%CI) = 1.35(1.17–1.57); *p* < .001] or those without functional dentition [HR(95%CI) = 1.34(1.17–1.55); *p* < .001].

**Conclusions:**

Having fewer NoT were associated with higher risks for all-cause mortality. More research is needed to explore possible biological implications and validate our findings.

**Supplementary Information:**

The online version contains supplementary material available at 10.1186/s12903-021-01934-0.

## Background

Tooth loss has been shown to correlate with several systemic comorbidities such as cardiovascular diseases, cancer, femoral neck bone mineral density or fractures [1–4]. Periodontal diseases, one of the major causes of tooth loss [5, 6], was found to be significantly associated with cardiovascular diseases [7], diabetes and the related healthcare expenditure [8, 9]. Recent reports have demonstrated that dental factors, such as the accumulation of oral health symptoms, or having severe tooth loss could serve as a significant predictor for mortality [10–13]. Elderly with less than functional dentition, weak chewing ability, or having difficulty in eating or swallowing later developed physical frailty (a geriatric syndrome manifested by fatigue, diminished strength, and reduced physical functioning leading to dependency or death [14]) and other adverse health outcome including mortality [11, 15]. Over the past decades, a decreasing trend of edentulism has been detected in the United States, although the improvement is not evenly distributed between the poor and the non-poor populations [16]. Indeed, socioeconomic status such as education and income levels that were highly correlated with access to dental care were demonstrated to be very predictive of tooth loss [17, 18]. Our goal for this study is to examine whether the number of remaining natural teeth (NoT), i.e., the severity of tooth loss, could be a predictor for the all-cause or disease-specific mortality in a representative general population. We used data from the United States National Health and Nutrition Examination Survey (NHANES) and its publicly linked mortality for this analysis.

It is noteworthy that when examining the NoT within a population, the distribution of NoT does not follow a common normal distribution and varies significantly depending on age and the study population. Therefore, to overcome the statistical challenges based on the distribution of NoT, different categories based on remaining dentition have been developed [1–3, 17–20]. In this manuscript, we used the WHO functional dentition (NoT 20 teeth or more) [17, 21, 22] as the criteria of the reference group of NoT20-28. Of note, NoT 20 is at the 75th percentile based on the decreasing NoT (with 28 teeth as the origin). We used NoT0-9 as the worst group, which contained the lowest14th percentile (one seventh) of the study samples (with edentulism as the origin). We then set NoT10-19 as the middle group (about 11%), which together with NoT0-9 dichotomized those participants with less complete dentition. The breakdown of NoT in the anterior or posterior segments of the mouth, or by the tooth types provided a more comprehensive way for understanding the patterns of tooth loss. Lastly, we used such other groupings of NoT as edentulism or functional dentition in the survival analysis in order to compare the differences in all-cause mortality risks given the severity of tooth loss.

## Methods

### Study design and population

The NHANES program under the Center for Disease Control and Prevention (CDC) uses a stratified, multistage, cluster sampling design to obtain a representative health and nutrition information of the U.S. household population. The NHANES consists of a detailed home interview and a health and dental examination conducted in a mobile examination center [23]. Starting from 1999, NHANES became a continuous (annual) program. Data from 1999 and after are publicly available (released every two years) with detailed survey operations and examination manuals on the website [24]. The NHANES program was approved by the United States National Center for Health Statistics Research Ethics Review Board and conforms to the STROBE guideline [25]. Our goal was to test whether the severity of tooth loss (as in NoT) could influence risks for the all-cause or disease-specific mortality. Here, we used the NHANES data from 1999 to 2014. Among 47,279 participants with mortality data, 7181 participants were excluded due missing dental information. An additional 7027 participants with any of the missing covariates except femoral neck bone mineral density were further excluded from the analysis, leaving 33,071 participants as the final sample size. We have provided the comparison of the excluded participants in Additional file 1: Table S1.

### Assessment of number of teeth present (NoT)

In the NHANES dental examination, tooth count is charted as primary, permanent, implant, missing or residual roots. Here, we used the sum of permanent dentition tooth count (excluding residual roots) as NoT. Third molars are excluded in the NHANES because of their frequent extraction in young adulthood. We did not include dental implants in the analysis because only a very low number of participants had dental implants (n = 379; 1.1%). Additionally, NoT was categorized into three groups based on the functional dentition [17–20], the 2nd lowest and the lowest seventh of the population based on NoT. The range of the NoT in three groups was as follows: NoT20-28, the reference group, (n = 24,770; 75%), NoT10-19 (n = 3653; 11%) and NoT0-9 (n = 4648; 14%). We also specify the edentulous (n = 2818; 8.5%) versus the dentate groups. In the supplementary information, we provide details of the distribution of the study population based on the total NoT (Additional file 1: Figure S1) and the patterns of tooth loss (breakdown of tooth types) based on age or the total NoT (Additional file 1: Table S2&3). In this analytical sample, the mean fraction of edentulism reached 5% when mean age was 48 years old. The mean number of molars fell below 1 when the total NoT was below 10.

### Assessment of mortality, hypertension, total cardiovascular diseases, diabetes and stroke

Mortality of the NHANES participants was ascertained by the National Center for Health Statistics with probabilistic record matching using the death certificates from the National Death Index, and converted to the public-use linked mortality file [26]. Vital status was determined form various sources, including through active follow-up of survey participants. Leading cause of death and other causes of interest were based on the ICD-10 (international statistical classification of diseases, 10th revision). Existing systemic comorbidities at the study interview such as total cardiovascular disease (CVD; including congestive heart failure, coronary heart disease, angina and heart attack) and stroke were summarized from the self-reported medical questionnaires [27, 28]. The presence of hypertension was defined with either of the following conditions—self-report of a physician’s diagnosis, the use of antihypertensive medications, or averaged blood pressure greater than 140/90 mmHg. Blood pressure readings were taken by a NHANES physician, usually 3 and sometimes 4 times. Diabetes mellitus was defined according to self-report of a physician’s diagnosis, the presence of a fasting plasma glucose level greater than 140 mg/dL or a random plasma glucose level greater than 200 mg/dL, or the use of diabetic medications (including insulin injection and oral hypoglycemic agents) [4, 27, 28]. For a subset of participants (n = 13,131), we included measurements of femoral neck bone mineral density (BMD; gm/cm^2^). Measurements of femoral neck BMD were obtained by utilizing dual-energy X-ray absorptiometry (DXA; Hologic Inc., Bedford, MA USA) [24] and were only available in the NHANES 2005–2010 and 2013–2014.

### Demographic data and other covariates

Age, sex, race and ethnicity, education levels, income to poverty ratios, and smoking status were obtained from the self-reported NHANES surveys. We categorized race/ethnicity into 4 groups (non-Hispanic white, African American, Hispanic and other), education in three levels (below high school, high school, and college and above), and smokers in 3 groups (never, past and current). Family income to the yearly poverty threshold issued by the Federal Register was developed as an index for determining financial eligibility for certain federal benefit programs. In this analysis, we used both education and the income-to-poverty ratio to account for socioeconomic status, which could alternatively infer the access to dental care [29] and tooth loss [17, 18]. Body mass index (BMI) was calculated as weight in kilograms divided by the square of height in meters from the NHANES body measures examination. Physical activities were obtained from questionnaires during the home interview and categorized as sedentary (5 h or more sedentary activities per day) [30], insufficient, moderate (moderate intensity activities 150 min or more per week), or vigorous (75 min or more vigorous-intensity aerobic activities or a combination of moderate and vigorous-intensity aerobic activities per week) as recent guideline suggested [31].

### Statistical analysis

The aim of this project was to assess the effect of NoT as represented by different groupings (independent variable) on the risks of all-cause or disease-specific mortality (dependent outcomes). In Tables 1 and 2, summary statistics are provided for participants based on their vital status. Comparisons were done by the non-parametric Kruskal–Wallis test for continuous variables and by chi-square tests for categorical variables to understand the differences between the living versus the deceased. In Table 3, characteristics were compared using similar tests as above across the three NoT groups (20–28, 10–19, and 0–9). In Table 4, we used Cox proportional hazard regression survival analyses in three levels of models to investigate the effect of NoT groups on risks of all-cause or disease-specific mortality. The time-to-event for survival analyses was calculated based on the interval between the linked death date in public record and the NHANES examination or interview dates. For disease specific mortality, we used the exclusive leading cause of death provided by the NHANES as the definition of mortality cases. Model 1 was adjusted for age, gender, race and smoking in 3 groups (never, past, and current). In Model 2, covariates such as education, income-to-poverty ratio, BMI, physical activities as well as the pre-existing medical conditions of hypertension, total cardiovascular diseases (CVD), diabetes, and stroke were further accounted for. In Model 3, the model was additionally controlled for measurements of femoral neck BMD, which decreased the available sample size due to data only available in specific years. All analyses were conducted in R statistical software and *P* values were set at < 0.05 for statistical significance.

**Table 1:** Characteristics of study participants based on all-cause mortality status

Groups^a^	Total	Alive	Deceased	
N	33,071	29,093	3978	*P* values ^b^
Age	49.32 (17.9)	46.67 (16.7)	68.72 (14.7)	< .001
Male	16,121 (48.7)	13,838 (47.6)	2283 (57.4)	< .001
*Race*				< .001
White	16,017 (48.4)	13,627 (46.8)	2390 (60.1)	
Black	6754 (20.4)	6028 (20.7)	726 (18.3)	
Hispanic	7986 (24.1)	7226 (24.8)	760 (19.1)	
Other	2314 (7.0)	2212 (7.6)	102 (2.6)	
*Education*				< .001
Below high school	8896 (26.9)	7225 (24.8)	1671 (42.0)	
High school or equivalent	7658 (23.2)	6677 (23.0)	981 (24.7)	
Above high school	16,517 (49.9)	15,191 (52.2)	1326 (33.3)	
IncPovR	2.59 (1.6)	2.64 (1.6)	2.22 (1.4)	< .001
BMI (kg/m^2^)	28.79 (6.6)	28.90 (6.7)	27.99 (6.2)	< .001
Femoral neck BMD^c^ (gm/cm^2^)	0.82 (0.15)	0.83 (0.1)	0.74 (0.2)	< .001
*Smoking*				
Never	17,581 (53.2)	15,962 (54.9)	1619 (40.7)	< .001
Past	8421 (25.5)	6862 (23.6)	1559 (39.2)	< .001
Current	7069 (21.4)	6269 (21.5)	800 (20.1)	0.042
*Physical activities*				< .001
Sedentary	15,959 (48.3)	13,959 (48.0)	2000 (50.3)	
Insufficient	4153 (12.6)	3470 (11.9)	683 (17.2)	
Moderate	7102 (21.5)	6143 (21.1)	959 (24.1)	
Vigorous	5857 (17.7)	5521 (19.0)	336 (8.4)	
*Systemic diseases*				
Total CVD	2672 (8.1)	1671 (5.7)	1001 (25.2)	< .001
Stroke	1126 (3.4)	689 (2.4)	437 (11.0)	< .001
Diabetes	4353 (13.2)	3316 (11.4)	1037 (26.1)	< .001
Hypertension	20,274 (61.3)	16,939 (58.2)	3335 (83.8)	< .001
*Cause of death*				
Maximum follow-up months	96.92 (54.4)	100.25 (54.6)	72.60 (46.2)	< .001
Heart diseases	686 (2.1)	NA	686 (17.2)	
Malignant neoplasms	920 (2.8)	NA	920 (23.1)	
Accidents	120 (0.4)	NA	120 (3.0)	
Cerebrovascular diseases	144 (0.4)	NA	144 (3.6)	
Diabetes MCOD	457 (1.4)	NA	457 (11.5)	

IncPovR, income-to-poverty ratio; BMI, body mass index; BMD, bone mineral density; CVD, cardiovascular disease; MCOD, flag from multiple cause of death

^a^Data are presented as mean (standard deviation) for continuous variables; and n (%) for categorical variables. ^b^*P*-values are from chi-square test for categorical variables or Kruskal–Wallis test for continuous variables in group comparisons. ^c^Data are available for 13,131 participants only

**Table 2:** Dental characteristics of study participants based on the all-cause mortality status

Groups^a^	Total	Alive	Deceased	
N	33,071	29,093	3978	*P* values ^b^
*Dental*				
NoT	21.52 (8.8)	22.60 (7.9)	13.68 (10.7)	< .001
Anterior NoT	10.06 (3.8)	10.49 (3.3)	6.90 (5.1)	< .001
Posterior NoT	11.47 (5.4)	12.11 (5.0)	6.78 (6.0)	< .001
Incisors	6.63 (2.6)	6.93 (2.3)	4.46 (3.5)	< .001
Canines	3.42 (1.2)	3.56 (1.1)	2.44 (1.7)	< .001
Premolars	6.08 (2.7)	6.38 (2.5)	3.89 (3.2)	< .001
Molars	5.38 (3.0)	5.72 (2.8)	2.88 (3.0)	< .001
NoT 20–28 (Functional)	24,770 (74.9)	23,149 (79.6)	1621 (40.7)	< .001
NoT 10–19	3653 (11.0)	2899 (10.0)	754 (19.0)	< .001
NoT 0–9	4648 (14.1)	3045 (10.5)	1603 (40.3)	< .001
Edentulous	2818 (8.5)	1738 (6.0)	1080 (27.1)	< .001

NoT, number of remaining natural teeth

^a^Data are presented as mean (standard deviation) for continuous variables; and n (%) for categorical variables. ^b^*P*-values are from chi-square tests for categorical variables or from Kruskal–Wallis tests for continuous variables in group comparisons

**Table 3:** Characteristics of study participants by number of teeth in NoT groups

Groups^a^	NoT 20–28	NoT 10–19	NoT 0–9	*P* value^b^
N (%)	24,770 (75)	3653 (11)	4648 (14)	
Age	44.21 (16.4)	61.38 (13.4)	67.09 (12.1)	< .001
Male	12,004 (48.5)	1801 (49.3)	2316 (49.8)	0.19
*Dental*				
NoT	26.03 (2.3)	15.37 (2.9)	2.37 (3.3)	< .001
Anterior	11.80 (0.7)	9.02 (2.4)	1.60 (2.4)	< .001
Posterior	14.23 (2.2)	6.36 (2.2)	0.77 (1.4)	< .001
Incisor	7.84 (0.6)	5.73 (2.0)	0.93 (1.6)	< .001
Canine	3.96 (0.2)	3.29 (0.8)	0.67 (1.0)	< .001
Premolar	7.38 (1.1)	4.26 (1.5)	0.62 (1.1)	< .001
Molar	6.85 (1.6)	2.09 (1.5)	0.15 (0.5)	< .001
*Smoking*				
Never	14,443 (58.3)	1574 (43.1)	1564 (33.6)	< .001
Past	5478 (22.1)	1160 (31.8)	1783 (38.4)	< .001
Current	4849 (19.6)	919 (25.2)	1301 (28.0)	< .001
*Cause of death*				
Total mortality	1621 (6.5)	754 (20.6)	1603 (34.5)	< .001
Maximum FU months	100.20 (54.6)	90.00 (52.6)	84.88 (52.0)	< .001
Heart diseases	238 (1.0)	131 (3.6)	317 (6.8)	< .001
Malignant neoplasms	389 (1.6)	174 (4.8)	357 (7.7)	< .001
Accidents	78 (0.3)	18 (0.5)	24 (0.5)	0.042
Cerebrovascular diseases	59 (0.2)	28 (0.8)	57 (1.2)	< .001
Diabetes MCOD	149 (9.2)	72 (9.5)	236 (14.8)	< .001
*Systemic*				
Hypertension	13,508 (54.5)	2882 (78.9)	3884 (83.6)	< .001
Total CVD	1175 (4.7)	503 (13.8)	994 (21.4)	< .001
Diabetes	2255 (9.1)	805 (22.0)	1293 (27.8)	< .001
Stroke	432 (1.7)	222 (6.1)	472 (10.2)	< .001
BMI (kg/m^2^)	28.71 (6.6)	29.41 (6.6)	28.69 (6.4)	< .001
Femoral Neck BMD^c^ (gm/cm^2^)	0.84 (0.1)	0.80 (0.2)	0.75 (0.1)	< .001

NoT, number of remaining natural teeth; FU, follow-up; MCOD, flag from multiple cause of death; CVD, cardiovascular disease; BMI, body-mass index; BMD, bone mineral density

^a^Data are presented as mean (standard deviation) for continuous variables; and n (%) for categorical variables. ^b^*P*-values are from chi-square test for categorical variables or Kruskal–Wallis test for continuous variables in group comparisons

**Table 4:** Risk of mortality based on NoT groups

Outcomes	Model 1	Model 2	Model 3
**All-cause mortality**	**HR (95% CI)**	**HR (95% CI)**	**HR (95% CI)**
N (event)	33,071 (3978)	33,071 (3978)	13,131 (1091)
NoT 20–28	Ref	Ref	Ref
NoT 10–19	1.33 (1.21–1.45)^***^	1.19 (1.09–1.31)^***^	1.23 (1.03–1.46)^*^
NoT 0–9	1.70 (1.58–1.84)^***^	1.37 (1.26–1.48)^***^	1.46 (1.25–1.71)^***^
	**HR (95% CI)**	**HR (95% CI)**	**HR (95% CI)**
Dentate	Ref	Ref	Ref
Edentulous	1.53 (1.42–1.64)^***^	1.24 (1.15–1.34)^***^	1.35 (1.17–1.57)^***^
	**HR (95% CI)**	**HR (95% CI)**	**HR (95% CI)**
NoT 20–28/Functional	Ref	Ref	Ref
NoT 0–19	1.54 (1.44–1.66)^***^	1.29 (1.20–1.39)^***^	1.34 (1.17–1.55)^***^
**Mortality (heart diseases)**			
N (event)	29,779 (686)	29,779 (686)	12,240 (200)
NoT 20–28	Ref	Ref	Ref
NoT 10–19	1.46 (1.18–1.82)^***^	1.29 (1.04–1.61)^*^	1.49 (0.98–2.26)
NoT 0–9	2.18 (1.82–2.61)^***^	1.59 (1.32–1.92)^***^	1.92 (1.33–2.77)^***^
**Mortality (diabetes)**			
N (event)	29,633 (540)	29,633 (540)	12,153 (113)
NoT 20–28	Ref	Ref	Ref
NoT 10–19	1.37 (1.06–1.77)^*^	1.06 (0.82–1.37)	0.97 (0.54–1.71)
NoT 0–9	2.64 (2.15–3.24)^***^	1.66 (1.35–2.05)^***^	1.67 (1.05–2.66)^*^
**Mortality (cancer)**			
N (event)	30,013 (920)	30,013 (920)	12,334 (294)
NoT 20–28	Ref	Ref	Ref
NoT 10–19	1.38 (1.14–1.66)^***^	1.33 (1.10–1.60)^**^	1.28 (0.91–1.81)
NoT 0–9	1.84 (1.57–2.15)^***^	1.63 (1.38–1.93)^***^	1.80 (1.34–2.43)^***^

Cox proportional hazard analysis was adjusted for age, gender, race and smoking in 3 groups in Model 1, and additionally adjusting for education (3 groups), income-to-poverty ratio, BMI, physical activities as well as the pre-existing medical conditions of hypertension, total CVD, diabetes, and stroke in Model 2. In Model 3, the model was additionally controlled for femoral neck BMD, which decreased the available sample size due to data availability

*P < 0.05; **P < 0.01; ***P < 0.001

NoT, number of remaining natural teeth; HR, hazard ratio; CI, confidence interval

## Results

### Characteristics of study participants based on the all-cause mortality status

There were 33,071 participants with available vital status, number of teeth from the dental examination, and complete information of the covariates (except that only 13,131 out of the 33,071 had measurements of femoral neck BMD). In Table 1, the deceased participants (before December 31, 2015) were older at the time of the NHANES interview and examinations. In the deceased group, there were more males (n = 2283, 57.4%), more white participants (n = 2390, 60.1%) and more past smokers (n = 1559, 39.2%) than that of the alive group. These deceased participants tended to have less education, lower income and less vigorous physical activities. There was also a higher prevalence of systemic comorbidities among the deceased at the time of the NHANES interviews, such as 25.2% of total CVD in this group versus 5.7% among those who survived. In the deceased group, 11% participants had histories of stroke, 26.1% had diabetes and 83.8% had hypertension; all were significantly higher than those of the living participants. Next, we investigated the distribution of the leading causes of death and whether there was a diabetes diagnosis flag from the multiple cause of death (MCOD). Among the 3978 deceased participants, 686 (17.2%) of them died of heart diseases, 920 (23.1%) of malignant neoplasms and 120 (3%) of accidents. There were 144 participants (3.6%) died of cerebrovascular diseases and 457 deaths (11.5%) flagged with diabetes MCOD. For the deceased participants, the mean BMI and femoral neck BMD were significantly lower compared to those who were alive.

### Dental characteristics based on the all-cause mortality status

Fewer NoT (mean 13.68) were found at the time of the NHANES dental examination among the later deceased participants, as compared to those survived (mean NoT = 22.6). When breaking down the NoT by segments or tooth types, there were less anterior teeth when comparing the later deceased vs. the living (mean 6.90 vs. 10.49), posterior teeth (mean 6.78 vs. 12.11), incisors (mean 4.46 vs. 6.93), canines (mean 2.44 vs. 3.56), premolars (mean 3.89 vs. 6.38) and molars (mean 2.88 vs. 5.72), respectively. More specifically, we noticed that there was a higher proportion of participants with NoT0-9 (40.3%) or being edentulous (27.1%) in the deceased group.

### Distribution of mortality, cause of death and dental characteristics based on the NoT groups

In Table 3, we present our analysis of the study participants based on the three NoT groups: NoT20-28, participants with functional dentition (i.e. having 20 or more teeth); NoT10-19, approximately the 2nd lowest seventh of this study population given the NoT; and NoT0-9, the lowest seventh based on the NoT. No difference was found in gender among the three NoT groups, but the mean age was older as the NoT decreased. Across the three groups with decreasing NoT, all-cause or disease specific mortality increased as the NoT decreased. Similarly, we noted that the prevalence of pre-existing systemic comorbidities (hypertension, total CVD, diabetes, and stroke) increased as the NoT decreased. For the measured femoral neck BMD, a significant decreasing trend along with the decrease of NoT was presented.

### Risk of all-cause and disease-specific mortality based on NoT in various groupings

In Table 4, we used Cox proportional hazard regression survival analyses to assess the effect of NoT as represented by different groupings on the risks of mortality. As shown in Model 1, NoT in the three groups or categorized as functional or edentulous were all significantly associated with all-cause mortality after adjusting for age, gender, race and smoking status. When compared to the reference group with functional dentition, risks for mortality were higher for those with NoT10-19 [HR(95%CI), 1.33(1.21–1.45); *p* < .001] and highest among those with NoT0-9 [HR(95%CI), 1.70(1.58–1.84); *p* < .001]. In Model 2, these effects remained comparable after further adjustment for risk factors such as education, income, BMI, and physical activities and the baseline systemic comorbidities (hypertension, total CVD, diabetes, and stroke). In Model 3, we additionally adjusted for the risk factor of femoral neck BMD, the hazard ratio of the NoT0-9 group remained significantly elevated in all-cause or disease specific mortality. The increase in risks of all-cause mortality for edentulous participants or those without functional dentition were also significant across three levels of models. Lastly, participants with NoT0-9 were at higher risks of death due to heart diseases [HR(95%CI), 1.92(1.33–2.77); *p* < .001], cancer [HR(95%CI), 1.80(1.34–2.43); *p* < .001], or diabetes [HR(95%CI), 1.67(1.05–2.66); *p* = 0.03]. We have provided detail outputs for the risks of all-cause mortality based on the NoT in 3 groups (Model 3) in the supplementary information (Additional file 1: Table S4 and Fig. S2).

## Discussion

In the present report, we demonstrated that the number of remaining natural teeth (NoT) is associated with all-cause and disease-specific mortality even after adjusting for traditional risk factors and baseline systemic comorbidities. Our results support prior research that the elderly who did not have posterior support [12, 13], who had fewer teeth [10] or who had a higher number of accumulated poor oral health symptoms [11] all demonstrated higher mortality rates compared to those with healthier oral conditions. Importantly, by using the NHANES data, we have further extended such associations between NoT and mortality in the context of the US community-dwelling population, after accounting for the baseline systemic comorbidities. The motivation to consider femoral neck BMD as a risk factor for all-cause mortality was based on a recent report [32], which similarly conducted analysis based on the NHANES data. Cai and colleagues identified that femoral neck BMD was predictive of the all-cause mortality after controlling for correlated risk factors. Here, in Table 4 Model 3, we tested and confirmed that such associations of lower NoT and higher risks of all-cause mortality remained significant after additional consideration of femoral neck BMD.

Both periodontal diseases and carious lesions are the primary reasons for tooth loss [5, 6]. Periodontal disease, a manifestation of processes involved in vascular endothelial dysfunction [33, 34], host immune vulnerability, inflammation [35, 36], and bone pathophysiology [37], eventually leads to loosening of teeth and tooth loss. On the other hand, stronger genetic components have been identified for carious lesions in recent consortium efforts [38]. We suggest that the number of remaining natural teeth should be considered as an important outcome variable for future research in dissecting and understanding the biology of oral health. Indeed, NoT captures tooth loss due to both periodontal diseases and carious lesions, both of which are genetically correlated [38]. Lastly, our findings support prior research of periodontal diseases and tooth loss associated with systemic comorbidities.

The strength of this study is the large sample size from the established NHANES surveys and examinations. On the other hand, given the inclusion requirement for this analysis (including completed dental examinations, with available mortality data, and non-missing information on covariates and systemic conditions), the authors acknowledge that there are limitations or potential bias due to the selection of the analytical samples. The excluded participants were younger (mean age 42.98 vs. 49.32), less white participants, more females and had higher mean NoT. There was also more total mortality or mortality due to heart disease, accidents or cerebrovascular diseases among those excluded. Therefore, our results cannot be translated to the general population without cautious interpretations. We are also aware that the NHANES examination weights for the general population were not used considering the sample consolidation process. Lastly, physical frailty [15] or late-life disability [27, 28] were found significantly associated with the number of remaining teeth or severe periodontitis. As life expectancy increases, active research has been devoted to understanding the severity of physical frailty and its implication in mortality and other outcomes [39, 40]. Many of the important risk factors for physical frailty were also included and adjusted for in this analysis. Of note, although the survival analyses demonstrated risk increase of all-cause mortality as the NoT decreased, causal inference shall not be implied based on our findings. Future research in a well-controlled prospective trial setting is needed to understand whether there is a potential mediating mechanism and the associated biological implications connecting NoT, physical frailty and mortality.

## Supplementary Information

**Additional file 1. Supplementary Table S1** Characteristics of excluded samples due to missing values. **Supplementary Table S2** Mean follow up time and number of teeth by age. **Supplementary Table S3** Pattern of tooth loss by total number of teeth (mean number of teeth by each tooth types). **Supplementary Table S4** Details of Cox proportional hazard analysis of all-cause mortality for the NoT in 3 groups (model 3). **Supplementary Figure S1** Distribution of number of teeth (NoT). **Supplementary Figure S2** Survival curves of all-cause mortality comparing three groups of NoT20-28, NoT10-19 and NoT0-9.

## Data Availability

NHANES data are available for public download through the website (https://wwwn.cdc.gov/nchs/nhanes/). The code files for processing this study are available from the corresponding author upon request.

## Abbreviations

NoT: Number of remaining natural teeth
NHANES: National Health and Nutrition Examination Survey in the United States
CDC: Center for Disease Control and Prevention
CVD: Cardiovascular disease
BMD: Bone mineral density
HR: Hazard ratio
CI: Confidence interval
