# Supplementary material for: Number of teeth is associated with all-cause and disease-specific mortality
Source: BMC Oral Health. 2021 Nov 8;21:568. doi: 10.1186/s12903-021-01934-0 (PMC8574051; doi:10.1186/s12903-021-01934-0)

Supplementary Table 1 Characteristics of excluded samples due to missing values

Out of a total of 47279 participants with survival data, 7181 did not complete dental examination. After excluding those without dental examination, additional 7027 participants with any of the missing covariates except femoral neck bone mineral density were further excluded from the analysis, leaving 33071 participants as the final sample size. Here we presented the demographics and mortality among those 14208 excluded participants as well as dental characteristics for hose excluded but with dental examinations.

Groups[a]		In the manuscript	
Total excluded	14208	33071	P Values [b]
Age	42.98 (22.7)	49.32 (17.9)	<.001
Male	6667 (46.9)	16121 (48.7)	<.001
**Race**			<.001
White	5427 (38.2)	16017 (48.4)	
Black	3286 (23.1)	6754 (20.4)	
Hispanic	4414 (31.1)	7986 (24.1)	
Other	1081 (7.6)	2314 (7.0)	
**Cause of death**			
Total mortality	2336 (16.4)	3978 (12.0)	<.001
Maximum follow-up months	98.35 (53.4)	96.92 (54.4)	0.006
Diseases of heart	426 (3.0)	686 (2.1)	<.001
Malignant neoplasms	412 (2.9)	920 (2.8)	0.49
Accidents	75 (0.5)	120 (0.4)	0.013
Cerebral vascular diseases	117 (0.9)	144 (0.4)	<.001
Diabetes MCOD	352 (10.9)	457 (11.5)	0.44
Excluded but with dental examination	7027	33071	
**Dental**			
NoT	22.90 (8.6)	21.52 (8.8)	<.001
Anterior NoT	10.37 (3.6)	10.06 (3.8)	<.001
Posterior NoT	12.54 (5.3)	11.47 (5.4)	<.001
Incisors	6.85 (2.5)	6.63 (2.6)	<.001
Canines	3.51 (1.2)	3.42 (1.2)	<.001
Premolars	6.47 (2.6)	6.08 (2.7)	<.001
Molars	6.07 (2.9)	5.38 (3.0)	<.001
NoT 20-28 (Functional)	5647 (80.4)	24770 (74.9)	<.001
NoT 10-19	532 (7.6)	3653 (11.0)	<.001
NoT 0-9	848 (12.1)	4648 (14.1)	<.001
Edentulous	559 (8.0)	2818 (8.5)	0.12

[a] Data are presented as mean (standard deviation) for continuous variables; and n (%) for categorical variables. [b] P-values are from chi-square test for categorical variables or Kruskal-Wallis test for continuous variables comparing groups.

Supplementary Table 2 Mean follow up time and number of teeth by age

Age	N (%)	Follow-up months	Number of teeth (NoT)	Anterior NoT	Posterior NoT	% of Edentulism
20	546 (1.65)	106.636	27.421	11.925	15.496	NA
21	587 (1.77)	109.806	27.354	11.894	15.46	0.17
22	557 (1.68)	109.032	27.329	11.916	15.413	NA
23	567 (1.71)	108.208	27.22	11.937	15.284	NA
24	569 (1.72)	106.747	27.186	11.896	15.29	0.351
25	519 (1.57)	106.489	27.079	11.865	15.214	0.193
26	536 (1.62)	115.61	26.935	11.834	15.101	0.56
27	496 (1.50)	110.635	26.768	11.786	14.982	0.806
28	506 (1.53)	110.466	26.686	11.739	14.947	0.395
29	559 (1.69)	113.644	26.508	11.794	14.714	0.537
30	618 (1.87)	101.052	26.471	11.723	14.748	0.485
31	631 (1.91)	101.452	26.338	11.685	14.653	0.951
32	610 (1.84)	105.161	26.272	11.77	14.502	0.492
33	576 (1.74)	101.934	25.842	11.628	14.214	0.694
34	617 (1.87)	103.196	25.943	11.635	14.308	0.972
35	560 (1.69)	106.134	25.918	11.604	14.314	1.25
36	619 (1.87)	98.861	25.538	11.488	14.05	1.292
37	560 (1.69)	107.434	25.575	11.525	14.05	0.714
38	601 (1.82)	100.027	25.304	11.499	13.805	1.165
39	603 (1.82)	101.907	24.94	11.396	13.544	1.161
40	655 (1.98)	102.252	24.73	11.371	13.359	2.443
41	596 (1.80)	99.416	24.97	11.349	13.621	1.678
42	565 (1.71)	104.977	24.773	11.414	13.359	1.239
43	600 (1.81)	100.655	24.242	11.19	13.052	2.5
44	618 (1.87)	100.942	23.676	11.008	12.668	3.074
45	612 (1.85)	103.268	24.255	11.263	12.992	2.288
46	594 (1.80)	102.487	23.379	10.946	12.433	2.862
47	553 (1.67)	100.544	23.541	11.033	12.508	2.712
48	530 (1.60)	96.821	22.483	10.509	11.974	5.849
49	541 (1.64)	98.085	22.342	10.523	11.819	6.285
50	586 (1.77)	94.684	22.309	10.575	11.734	4.949

51	585 (1.77)	98.974	21.665	10.407	11.258	5.128
52	561 (1.70)	96.036	21.203	10.091	11.112	5.526
53	523 (1.58)	96.403	21.306	10.256	11.05	6.501
54	545 (1.65)	100.49	21.002	10.106	10.895	6.422
55	482 (1.46)	88.566	20.214	9.793	10.421	6.846
56	466 (1.41)	92.3	20.552	9.807	10.745	7.296
57	417 (1.26)	91.312	20.782	9.959	10.823	8.873
58	380 (1.15)	90.942	18.479	9.129	9.35	13.158
59	401 (1.21)	88.369	20.738	9.908	10.83	6.484
60	700 (2.12)	96.661	19.196	9.46	9.736	9.143
61	614 (1.86)	94.878	18.073	8.873	9.2	12.704
62	600 (1.81)	95.142	17.693	8.688	9.005	13.667
63	569 (1.72)	89.476	17.058	8.571	8.487	14.06
64	499 (1.51)	94.926	17.084	8.543	8.541	14.028
65	530 (1.60)	92.753	16.919	8.487	8.432	14.528
66	481 (1.45)	93.025	16.372	8.168	8.204	17.464
67	437 (1.32)	92.375	16.801	8.387	8.414	16.934
68	426 (1.29)	93.831	15.962	7.993	7.969	16.432
69	386 (1.17)	91.241	15.161	7.642	7.518	20.984
70	475 (1.44)	93.545	15.299	7.762	7.537	20.842
71	420 (1.27)	88.795	14.102	7.238	6.864	24.524
72	400 (1.21)	85.597	14.408	7.268	7.14	25
73	398 (1.20)	91.812	14.93	7.585	7.344	20.101
74	353 (1.07)	86.912	13.748	7.042	6.705	28.045
75	343 (1.04)	83.872	13.131	6.834	6.297	26.531
76	322 (0.97)	84.189	13.071	6.668	6.404	29.193
77	263 (0.80)	85.62	13.859	7.194	6.665	24.335
78	282 (0.85)	79.394	14.106	7.266	6.84	22.695
79	235 (0.71)	75.757	12.889	6.762	6.128	28.511
80	1174 (3.55)	56.311	13.124	6.762	6.361	27.683
81	180 (0.54)	96.039	14.061	7.133	6.928	26.111
82	128 (0.39)	97.617	14.57	7.359	7.211	18.75
83	103 (0.31)	90.301	14.272	7.32	6.951	29.126
84	101 (0.31)	78.871	13.554	7.099	6.455	26.733
85	405 (1.22)	70.76	10.568	5.494	5.074	38.519

Supplementary Table 3 Pattern of tooth loss by total number of teeth (mean number of teeth by each tooth types)

NoT	N (%)	Follow-up months	Incisor	Canine	Premolar	Molar
28	10403 (31.46)	101.878	8	4	8	8
27	3386 (10.24)	102.944	7.909	3.969	7.798	7.324
26	2760 (8.35)	101.3	7.827	3.946	7.491	6.737
25	1844 (5.58)	97.377	7.803	3.935	7.266	5.996
24	2402 (7.26)	100.831	7.787	3.935	5.97	6.308
23	1344 (4.06)	95.324	7.571	3.911	6.592	4.926
22	1040 (3.14)	94.313	7.466	3.896	6.297	4.34
21	845 (2.56)	93.318	7.265	3.858	6.05	3.827
20	746 (2.26)	90.084	7.194	3.834	5.631	3.34
19	649 (1.96)	92.636	7.025	3.77	5.344	2.861
18	509 (1.54)	87.778	6.766	3.715	4.994	2.525
17	419 (1.27)	90.986	6.408	3.568	4.69	2.334
16	439 (1.33)	91.613	5.993	3.481	4.394	2.132
15	310 (0.94)	83.826	5.59	3.439	4.168	1.803
14	278 (0.84)	85.737	5.212	3.191	3.694	1.903
13	230 (0.70)	91.857	4.887	3.009	3.361	1.743
12	274 (0.83)	89.847	4.376	2.759	3.252	1.613
11	246 (0.74)	94.13	4.008	2.427	3.114	1.451
10	299 (0.90)	90.007	3.716	2.187	3.064	1.033
9	272 (0.82)	87.882	3.555	2.059	2.728	0.658
8	335 (1.01)	89.701	3.343	2.03	2.179	0.448
7	265 (0.80)	91.777	3.015	1.928	1.649	0.408
6	304 (0.92)	86.155	2.76	1.839	1.122	0.28
5	172 (0.52)	81.215	1.75	1.477	1.448	0.326
4	152 (0.46)	80.013	1.086	1.388	1.211	0.316
3	136 (0.41)	81.279	0.676	1.132	0.963	0.228
2	139 (0.42)	86.871	0.209	1.194	0.489	0.108
1	55 (0.17)	78.582	0.145	0.491	0.236	0.127
0	2818 (8.52)	83.92	0	0	0	0

Supplementary Table 4

Details of Cox proportional hazard analysis of all-cause mortality for the NoT in 3 groups (Model 3)

N= 13131, Event= 1091	HR (95% CI)	P value
NoT 20-28	**Ref.**	
NoT 10-19	***1.23 (1.03-1.46)***	***0.019***
NoT 0-9	***1.46 (1.25-1.70)***	***1.40e^{-06}***
Other covariates		
Age	***1.07 (1.06-1.07)***	***1.06e^{-95}***
Male	***1.71 (1.49-1.96)***	***1.55e^{-14}***
Race (Black)	0.96 (0.81-1.14)	0.651
Race (Hispanic)	***0.81 (0.68-0.98)***	***0.027***
Race (Other)	***0.69 (0.48-0.99)***	***0.044***
Past smokers	1.04 (0.91-1.20)	0.557
Current smokers	***1.47 (1.23-1.76)***	***1.77e^{-05}***
BMI	***0.97 (0.95-0.98)***	***1.07e^{-06}***
High school or equivalent	0.97 (0.83-1.13)	0.697
Above high school	0.91 (0.78-1.07)	0.253
Income-to-poverty ratio	***0.84 (0.80-0.88)***	***1.64e^{-12}***
Exercise (Insufficient)	***0.78 (0.64-0.93)***	***0.007***
Exercise (Moderate)	***0.61 (0.53-0.72)***	***7.44e^{-10}***
Exercise (Vigorous)	***0.64 (0.51-0.80)***	***1.16e^{-04}***
Total CVD	***1.40 (1.21-1.62)***	***8.69e^{-06}***
Diabetes	***1.52 (1.32-1.75)***	***7.62e^{-09}***
Stroke	***1.54 (1.27-1.87)***	***9.82e^{-06}***
Hypertension	1.13 (0.95-1.34)	0.168
Femoral neck BMD	***0.35 (0.21-0.59)***	***8.43e^{-05}***

Abbreviations: NoT, number of teeth; CVD, cardiovascular diseases; BMI, body-mass index; BMD, bone mineral density.

Suppl. Figure 1 Distribution of NoT

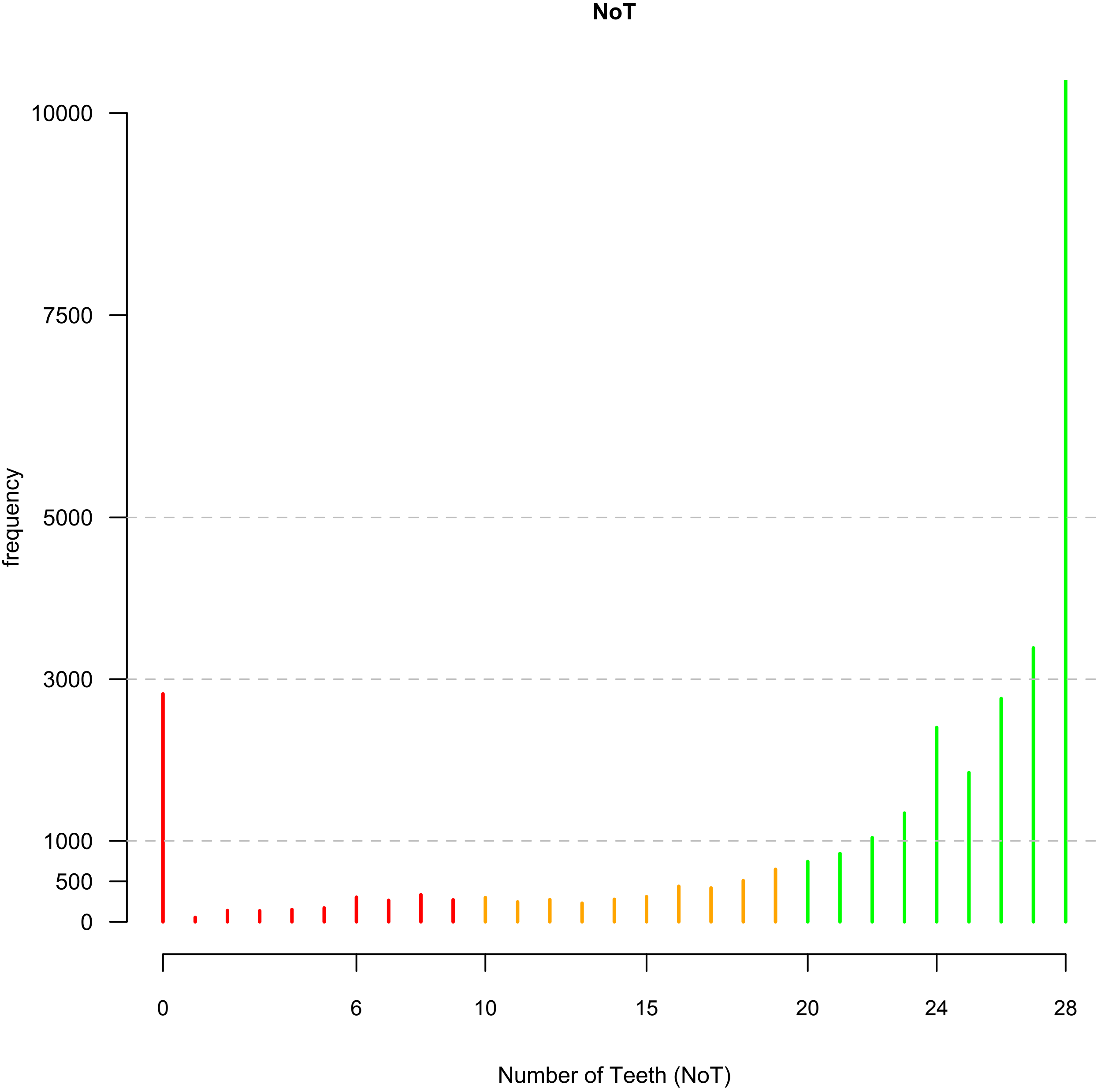

Suppl. Figure 2

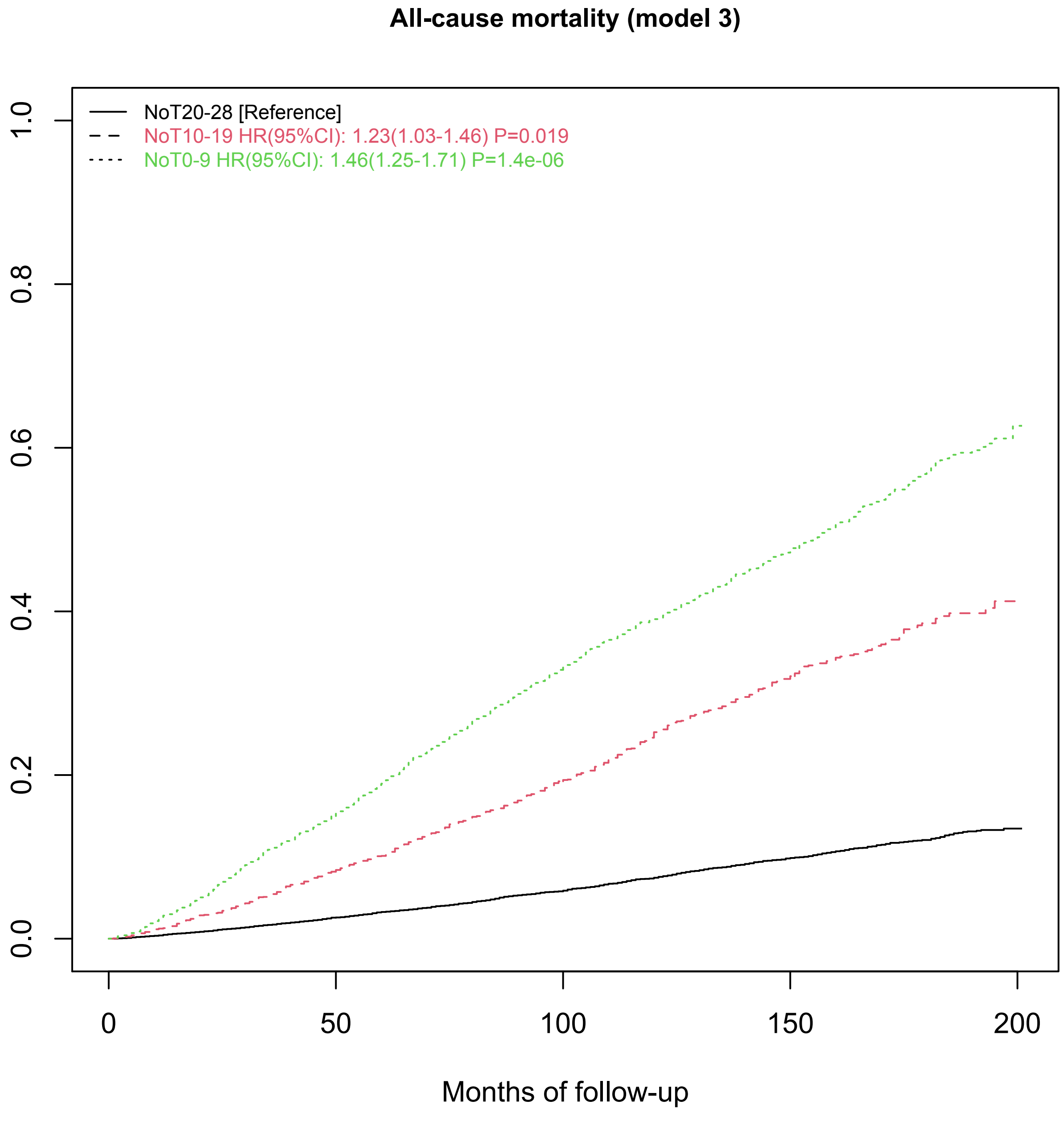

Supplement: Supplementary file 1 — Additional file 1. Supplementary Table S1 Characteristics of excluded samples due to missing values. Supplementary Table S2 Mean follow up time and number of teeth by age. Supplementary Table S3 Pattern of tooth loss by total number of teeth (mean number of teeth by each tooth types). Supplementary Table S4 Details of Cox proportional hazard analysis of all-cause mortality for the NoT in 3 groups (model 3). Supplementary Figure S1 Distribution of number of teeth (NoT). Supplementary Figure S2 Survival curves of all-cause mortality comparing three groups of NoT20-28, NoT10-19 and NoT0-9. [file 12903_2021_1934_MOESM1_ESM.pdf]
